# Amyloid-β Protofibrils: Size, Morphology and Synaptotoxicity of an Engineered Mimic

**DOI:** 10.1371/journal.pone.0066101

**Published:** 2013-07-02

**Authors:** Anatoly Dubnovitsky, Anders Sandberg, M. Mahafuzur Rahman, Iryna Benilova, Christofer Lendel, Torleif Härd

**Affiliations:** 1 Department of Molecular Biology, Swedish University of Agricultural Sciences (SLU), Uppsala, Sweden; 2 Alzinova AB, Gothenburg, Sweden; 3 Department for Molecular and Developmental Genetics, Flanders Institute for Biotechnology (VIB), Leuven, Belgium; 4 Center for Human Genetics, KULeuven, Leuven, Belgium; Cambridge Institute for Medical Research, United Kingdom

## Abstract

Structural and biochemical studies of the aggregation of the amyloid-β peptide (Aβ) are important to understand the mechanisms of Alzheimer's disease, but research is complicated by aggregate inhomogeneity and instability. We previously engineered a hairpin form of Aβ called Aβcc, which forms stable protofibrils that do not convert into amyloid fibrils. Here we provide a detailed characterization of Aβ_42_ cc protofibrils. Like wild type Aβ they appear as smooth rod-like particles with a diameter of 3.1 (±0.2) nm and typical lengths in the range 60 to 220 nm when observed by atomic force microscopy. Non-perturbing analytical ultracentrifugation and nanoparticle tracking analyses are consistent with such rod-like protofibrils. Aβ_42_ cc protofibrils bind the ANS dye indicating that they, like other toxic protein aggregates, expose hydrophobic surface. Assays with the OC/A11 pair of oligomer specific antibodies put Aβ_42_ cc protofibrils into the same class of species as fibrillar oligomers of wild type Aβ. Aβ_42_ cc protofibrils may be used to extract binding proteins in biological fluids and apolipoprotein E is readily detected as a binder in human serum. Finally, Aβ_42_ cc protofibrils act to attenuate spontaneous synaptic activity in mouse hippocampal neurons. The experiments indicate considerable structural and chemical similarities between protofibrils formed by Aβ_42_ cc and aggregates of wild type Aβ_42_. We suggest that Aβ_42_ cc protofibrils may be used in research and applications that require stable preparations of protofibrillar Aβ.

## Introduction

Alzheimer's disease (AD) is associated with an imbalance in the production and clearance of the amyloid-β peptide (Aβ) followed by Aβ aggregation in the brain [1]. The aggregation ultimately ends in the formation of insoluble protein fibrils as components of amyloid plaques. Considerable evidence suggests that neurotoxic species are soluble oligomers or protofibrils of Aβ that are present on or off aggregation pathways leading to fibril formation [2], [3], [4], [5], [6], [7], [8]. The 42-residue Aβ_42_ fragment is in this regard more aggregation prone than the more prevalent but less active Aβ_40_ fragment and an increase in the Aβ_42_: Aβ_40_ ratio is also associated with increased neurotoxicity [9]. Other evidence suggests that the rate of aggregation, and not only the aggregates that are present, acts to further enhance toxicity [10], [11].

Aβ can form a multitude of interconverting toxic aggregates both *in vitro* and *in vivo* [12], [13], [14]. However, in all cases, aggregate inhomogeneity and instability complicate research on correlations between aggregation, structure and toxicity. Different ways to stabilize intermediate aggregates by chemical cross linking [for instance [6]] or protein engineering [[15] and work cited therein] have therefore been devised.

We recently engineered a double cysteine mutant of Aβ (Aβcc) for which aggregation is halted at the protofibrillar state [16], which is suggested to be the penultimate intermediate prior to amyloid fibril formation [13], [14], [17]. Briefly, Aβcc was designed to test a structural model of aggregation [18] in which Aβ adopts a hairpin conformation in aggregates on the path to fibril formation [18]. This model hypothesized that a conformational change in such aggregates results in the formation of seeds for runaway fibril polymerization. Aβcc contains a double Ala21Cys/Ala30Cys mutation and a disulfide bond formed between the two cysteines locks the peptide into the hairpin conformation [16]. Aβcc should therefore, according to the proposed model, form soluble oligomers and protofibrils, but be unable to adopt the cross-β structure present in mature fibrils. We found that this indeed is the case: Aβcc can readily form oligomeric species, which do not convert into fibrils unless the intramolecular disulfide bond is broken by reduction [16]. Protofibrils of Aβ_42_ cc are stable towards both dissociation (upon dilution) and fibril formation.

More importantly, protofibrils formed by Aβ_42_ cc potentially constitute a tool for experiments in which stable protofibrils are required, such as for instance structural studies or immunization trials. Initial studies indicated a number of similarities between wild type and Aβ_42_ cc protofibrils [16]. Here we characterize Aβ_42_ cc protofibrils in more detail, and compare them to wild type protofibrils, with regard to their morphology and size, surface properties and exposed antibody epitopes, protein binding, and their effect on synaptic activity in neurons.

## Materials and Methods

### Protein production and preparation of aggregates

Aβ_40_, Aβ_42_ and Aβ_42_ cc were produced in *E. coli* by co-expression with the ZAβ3 Aβ-binding Affibody® molecule as described previously [16], [19]. The peptides were separated from the Affibody binder by denaturation in 7 M guanidinium chloride followed by immobilized metal affinity chromatography (IMAC) under denaturing conditions. Aβ_42_ cc protofibrils were obtained by separating oligomers with size exclusion chromatography (SEC) under native buffer conditions [16], followed by concentration on a Vivaspin column (GE Healthcare) and heat treatment at 60°C for 10 min. Alternatively, protofibrils also form when guanidinium chloride is removed by dialysis of denatured Aβ_42_ cc at room temperature against 20 mM sodium phosphate buffer at pH 7.2 with 50 mM NaCl and 5 mM EDTA and a second dialysis for 4 to 6 hours in the same buffer without EDTA.

Protofibrils of wild type Aβ_42_ were identified in atomic force microscopy (AFM) images of an Aβ_42_ aggregation reaction mixture. Aβ_42_ monomer (∼100 μM) in 20 mM sodium phosphate buffer at pH 7.2 with 50 mM NaCl was kept at room temperature without shaking. A mixture of Aβ_42_ protofibrils and smaller oligomers could be observed and distinguished in AFM images recorded after one day of incubation. The same solution was put in 37°C and shaking conditions for one more day in order to produce Aβ_42_ fibrils for AFM imaging. Fibrils of wild type Aβ_40_ and Aβ_42_ for AFM and OC serum dot blot assays were prepared from similar mixtures that were subjected to 37°C and shaking to favor the formation of fibrils.

### Atomic force microscopy

Concentrated protofibrils or fibrils of Aβ_42_ cc, Aβ_42_, or Aβ_40_ were diluted to 0.5 to 1 µM in 20 mM sodium phosphate buffer at pH 7.2 with 50 mM NaCl, and 5 µL solutions were loaded onto freshly cleaved mica. After 1 to 2 min, the mica surface was briefly washed with 100 µL deionized water and air-dried. The samples were imaged immediately in AC-mode using a Cypher AFM instrument (Asylum Research, USA) equipped with NSC36/Si3N4/AlBs three-lever probes (µMasch). The probes had nominal spring constants of 0.6 to 1.8 N/m and driving frequencies of 75 to 155 kHz. To determine protofibril length distributions, a number of images covering 1 to 2 µm^2^ surfaces were scanned and the lengths of particles were measured using a freehand tool in the MFP-3D™ offline section analysis software. The same tool was used to measure cross sections of particles.

### Analytical ultracentrifugation

Sedimentation velocity data were collected using the UV-visible optics detector on a Beckman Optima XL-A centrifuge equipped with an An-60Ti 4-cells rotor and double-sector 12 mm Epon centerpieces with quartz windows. The measurements were carried out at 17,000 rpm and 20°C. The Aβ_42_ cc protofibril concentration was 300 µM (monomer) in 20 mM sodium phosphate buffer at pH 7.2 with 50 mM NaCl and 0.05% NaN_3_. Absorption was recorded at 280 nm and sedimentation profiles were collected every 5 min. Data were analyzed using the SEDFIT program (v 12.52; http://analyticalultracentrifugation.com/default.htm) [20] using continuous distributions of Lamm-equation solutions with a common (unimodal) frictional ratio. The partial specific volume of Aβ_42_ cc, and the buffer density and viscosity at 20°C, were calculated using the SEDNTERP program v 1.09 (Biomolecular Interaction Technologies Center, University of New Hampshire, Durham, NH; http://bitcwiki.sr.unh.edu/index.php/Main_Page). Measured sedimentation coefficients were corrected to *s_20, w_* values (sedimentation coefficients in water at 20°C).

### Nanoparticle tracking analysis (NTA)

Measurements were performed with a NanoSight LM10-HS instrument (NanoSight, Amesbury, UK) equipped with an LM14 viewing unit using a 405 nm laser. Protofibrils of Aβ_42_ cc were diluted in 20 mM sodium phosphate buffer at pH 7.2 with 50 mM NaCl to a final monomer concentration of 2 µM, and measurements were performed at 20°C for 30 s. The NanoSight NTA 2.2 software was used for data analysis.

### OC serum dot blot

The anti-amyloid fibril OC rabbit serum (Millipore) [21] was used at 1∶1000 dilution according to the manufacturer's instructions. Samples were diluted to 5 µM monomer concentrations and 2.5 µL of each sample was loaded onto untreated cellulose nitrate Protran BA85 membranes (Schleicher & Schuell, Germany) and allowed to dry. An HRP-conjugated goat anti-rabbit IgG antibody (H+L, Invitrogen) was used to detect bound OC antibodies using chromogenic 3,3',5,5'-tetramethylbenzidine (Novex®, Invitrogen) as substrate.

### Synaptotoxicity

The effect of Aβ_42_ cc protofibrils on spontaneous synaptic activity was evaluated in an *in vitro* microelectrode array (MEA) assay [9]. Soluble oligomers of Aβ_42_ were used for comparison. These were prepared as described previously (Ref. [9]; the 10∶0 Aβ_42_: Aβ_40_ ratio oligomers), with the modification that a 20 mM sodium phosphate buffer at pH 7.2 with 50 mM NaCl was used to match the Aβ_42_ cc buffer. Primary hippocampal neurons were dissected from e17 FVB mouse embryos and plated on MEA substrate (Multichannel Systems GmbH, Germany) at a density of 1000 cells mm^−2^ (500,000 cells per chip). The spontaneous firing of neuronal networks was recorded after 1 to 2 weeks in culture. A temperature controller (Multichannel Systems) was used to maintain the MEA platform temperature at 37°C during the experiments. First, the basal firing rate was recorded for 500 s, then 0.5 µM of either Aβ_42_ oligomers or Aβ_42_ cc protofibrils was added to MEA dish and neuronal activity was recorded for the next 500 s. The same amounts of Aβ was added two more times to reach final concentration of 1.5 µM. Signals from active electrodes were amplified by means of a MEA1060 amplifier (gain 1200) and digitized by the A/D MC_Card at a sampling rate of 25 kHz. The MC_Rack 3.5.10 software (Multichannel Systems) was used for data recording and processing. The raw data were high-pass filtered at 200 Hz, and the threshold for spike detection was set to 5 standard deviations from the average noise amplitude computed during the first 1000 ms of recording. Numbers of spikes detected by every active electrode per time bin of 500 s were normalized to baseline (firing rate in the absence of treatment). The firing rates corresponding to 500 s treatments with 0.5, 1 and 1.5 µM of protofibrils/oligomers were computed and presented as percentage of initial rates.

Use of animals and procedures were approved by the Ethical Committee for Animal Welfare (ECD, Ethische Commissie Dierenwelzijn) of KULeuven and IMEC. Timely pregnant FVB mice were sacrificed with CO_2_, and the embryos were removed immediately thereafter.

### Fluorescence spectroscopy

Fluorescence emission spectra of peptide-ANS mixtures were recorded at room temperature on a Varian Cary Eclipse spectrofluorometer using a 0.3 cm path length quartz cuvette and an excitation wavelength of 360 nm. Aβ_42_ cc monomer samples were obtained as the monomeric fraction in SEC, concentrated and kept frozen until use. Aβ_42_ cc monomer and protofibril solutions both contained 10 µM peptide in 20 mM sodium phosphate buffer at pH 7.2, with 50 mM NaCl. The ANS concentration was 50 µM.

### Binding to serum proteins

Aβ_42_ cc protofibrils were immobilized on tosyl-activated M280 Dynabeads (Invitrogen) according to the manufacturer's protocol. Briefly, 5 mg of beads were incubated with 100 µg of Aβ_42_ cc protofibrils in 0.1 M sodium phosphate buffer, pH 7.4 overnight at 37°C to allow covalent binding of Aβ_42_ cc to the beads. The beads were then washed with PBS buffer with 0.5% Tween-20. As control, glycine was immobilized to the same type of beads. 0.5 mg coupled Dynabeads was then incubated with 150 μL human serum (3H Biomedical, Uppsala) for 1 h at 37°C and then washed three times. Bound proteins were eluted using SDS-PAGE sample buffer and separated using SDS-PAGE (4–20% gradient gel from BioRad). The bands were visualized using Acquastain (Acquascience, USA). Separated gel bands were cut, destained in 30% ethanol, trypsin-digested and subjected to mass spectrometry analysis using an Ultraflex II MALDI TOF mass spectrometer (Bruker Daltonics, Bremen, Germany). Proteins were identified using the Mascot search engine (www.matrixscience.com) [22].

## Results and Discussion

### Preparation and stability of Aβ_42_cc protofibrils

With the terminology used here, oligomers are soluble aggregates that can be separated by size exclusion chromatography. The most abundant of the Aβ_42_ cc oligomers is a β-sheet containing aggregate with an apparent MW of 100 kDa [16]. Protofibrils are much larger aggregates that are clearly rod-like and with an apparent AFM *z*-height of 3.1 nm, as described below. We previously prepared protofibrils of Aβ_42_ cc by concentrating the β-sheet-containing oligomers that form when guanidinium chloride solutions are diluted into non-denaturing buffer conditions during size exclusion chromatography [16]. A more direct way to obtain Aβ_42_ cc protofibrils is by removal of guanidinium chloride *via* dialysis (see *Materials and Methods*). The biophysical properties of Aβ_42_ cc protofibrils obtained by these two different methods are not distinguishable. However, the dialysis method results in two to three fold higher final yield of protofibrils while being less laborious. Therefore, the Aβ_42_ cc protofibrils used in the experiments described below were obtained using the dialysis method. Preparations of Aβ_42_ cc protofibrils are stable towards dissociation when diluted with buffer, while smaller Aβ_42_ cc oligomers may dissociate into monomeric species upon dilution (A. Sandberg and A. Dubnovitsky, unpublished). Protofibril preparations are also stable towards dissociation for months at room temperature.

### Protofibril size and rod-like morphology revealed by AFM

We used atomic force microscopy (AFM) to study the size and morphology of Aβ_42_ cc particles on dry mica surface. Protofibrils are obtained in 20 mM sodium phosphate buffer with 50 mM NaCl and sample preparation for AFM in air requires removal of salts. This may be achieved by diluting concentrated protofibril solutions in deionized water before loading them onto mica. However, with such a procedure only smaller Aβ_42_ cc protofibrils appear to bind the mica. Therefore, special care was taken to minimize exposure to low-salt environment.

**Figure 1:**
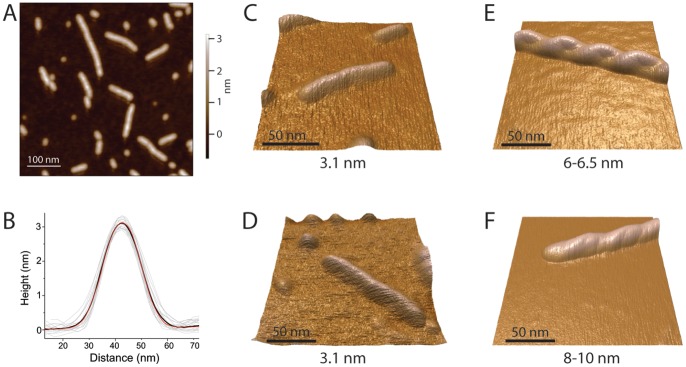
Analysis of Aβ_42_ cc morphology using atomic force microscopy (AFM). (A) AFM image of Aβ_42_ cc protofibrils on dry mica surface. (B) Average *z*-heights and cross-sections of Aβ_42_ cc (black) and wild type Aβ_42_ (red) protofibrils (grey lines represent measurements of 20 Aβ_42_ cc protofibrils). (C-F) High magnification AFM images of single protofibrils of Aβ_42_ cc (C) and wild type Aβ_42_ (D; identified in aggregation reaction mixtures, Fig. S2), and of amyloid fibrils of Aβ_40_ (E) and Aβ_42_ (F). Measured *z*-heights of particles are indicated in panels C-F.

Purified protofibrils were diluted in buffer, loaded onto freshly cleaved mica, and briefly washed with water. Prepared in this way, Aβ_42_ cc protofibrils appear in AFM as unbranched rod-like structures with a well defined average height of 3.1 (±0.16) nm and typical lengths in the range 50 to 220 nm (Fig. 1A–B). The largest protofibril is more than 600 nm long (Fig. S1). The protofibril width measured here (AFM *z*-height) is smaller than widths of wild type Aβ protofibrils [23], [24] and Aβ_42_ cc protofibrils [16] observed in electron microscopy with uranyl acetate negative staining (6 to 10 nm), but a 3.1 nm height was also reported in a detailed AFM analysis of protofibrils of wild type Aβ_40_ [25].

High-resolution scans of single protofibril particles reveal a smooth surface without distinguishing features (Fig. 1C). The shape and size of Aβ_42_ cc protofibrils are identical to those of wild type Aβ_42_ protofibrils identified in aggregation reaction mixtures of 100 µM peptide stored at room temperature without shaking (Fig. 1B–D, Fig. S2). The morphology of Aβ_42_ cc and Aβ_42_ protofibrils are both very different from the characteristic helical appearance of Aβ_40_ or Aβ_42_ fibrils imaged under the same conditions (Fig. 1E–F). (The dimension and morphology of the Aβ_40_ fibril are such that it may be envisioned as a double helix of 3.1 nm filaments.)

**Figure 2:**
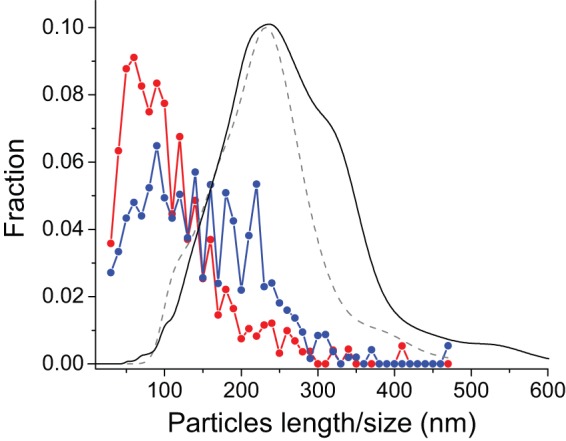
Size distribution of Aβ_42_ cc protofibrils measured using different methods. The blue and red lines/symbols represent data from atomic force microscopy. One sample (blue) was washed briefly with deionized water, while a second sample (red) was washed extensively. The lengths of *ca.* 1500 protofibrils were measured in each case. The gray dashed line reflects an expected distribution corresponding to the analytical ultracentrifugation measurements (Fig. 3) assuming that Aβ_42_ cc protofibrils have a dehydrated diameter of 3.1 nm. The black line represents the distribution of apparent hydrodynamic radius obtained from nanoparticle tracking analysis using a NanoSight microscope.

Short Aβ_42_ cc protofibrils appear to be straight in AFM, but smooth curvatures can be observed with increasing particle length. The precise persistence length of the rods was, however, not determined. If protofibrils bound to mica are extensively washed with water, more sharp kinks appear together with multiple brakes in long protofibrils. We characterized the protofibril length distributions in two different samples of Aβ_42_ cc protofibrils (Fig. 2). One sample was washed only very briefly with water, as above, while the other was washed extensively several times. Extensive washing with water disrupts the protofibrils (on mica) and many particles become shorter than 100 nm (Fig. 2). Interestingly, the two size distributions show a similar fine structure (or periodicity) indicating that protofibril disruption involves dissociation of discrete oligomeric building blocks.

### Protofibril size distribution in solution

**Figure 3:**
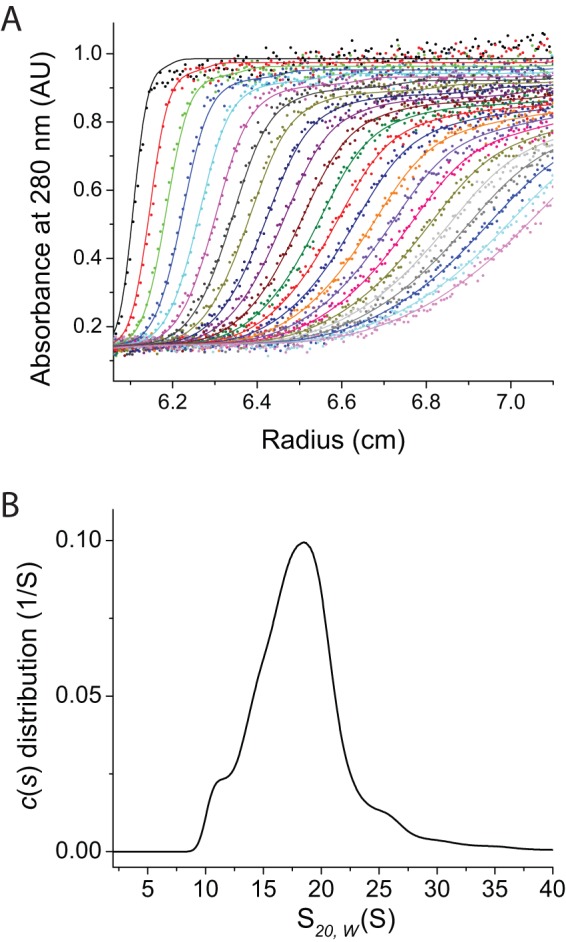
Size distribution of Aβ_42_ cc protofibrils in solution monitored by analytical ultracentrifugation. (A) A subset of the raw sedimentation velocity centrifugation data of 300 µM Aβ_42_ cc protofibrils at 20°C recorded over a period of 20 h. (B) Sedimentation coefficient distribution of Aβ_42_ cc protofibrils analyzed using a continuous c(*s*) distribution model.

The size distribution of Aβ_42_ cc protofibrils in solution was analyzed by non-invasive analytical ultracentrifugation (AUC). Data analysis assuming a continuous c(*s*) distribution model indicates a distribution of large particles between 2 and 30 S (Fig. 3). A size distribution centered at *s_20, w_* = 18 S with a best-fit frictional ratio *f*/*f_0_* of 2.7 to 3.1 suggests the presence of long rod-like particles. For comparison, *f*/*f_0_* = 2.3 and *s_20, w_* = 7.6 for human fibrinogen (MW  = 330,000 Da), and *f*/*f_0_* = 3.6 and *s_20, w_* = 6.4 for myosin (MW  = 570,000 Da) [26].

A simple model calculation in which protofibrils rods are assumed to be 3.1 nm in diameter and hydrated suggests that the average *s_20,w_* = 18 S corresponds to a length of 220 to 230 nm, which is somewhat longer than the average length observed with AFM (Fig. 2). Hence, it is likely that sample preparation for AFM measurements results in protofibril breakage, which is also consistent with the observation that washing with deionized water results in shorter protofibrils. A theoretical length distribution derived from the AUC data is shown together with the AFM lengths in Fig 2.

We also studied the size distribution of Aβ_42_ cc protofibrils in solution using nanoparticle tracking analysis (NTA) using a NanoSight microscope in which laser light scattering allows for tracking of the Brownian motion of individual nanoparticles. The hydrodynamic radius is then determined using the Stokes-Einstein equation based on the mean square speed of a particle. This technique is particularly valuable for analyzing polydisperse nanosized particles [27]. The size distribution of Aβ_42_ cc protofibrils obtained from NTA (Fig 2, solid black line) shows that most particles are found in the range of 100–400 nm. This result is in good agreement with the length distribution calculated from AUC data using a dehydrated particles height obtained from AFM measurements (Fig. 2, dashed grey line). Thus, using two independent methods we demonstrate similar size distribution of Aβ_42_ cc protofibrils in solution with an average length of 220 to 230 nm.

### ANS binding to Aβ_42_cc protofibrils reveals hydrophobic surface patches

1-anilinonaphtalene 8-sulfonic acid (ANS) is a fluorescent dye that is widely used to probe the presence of exposed hydrophobic patches or cavities on proteins [28], [29]. Bolognesi *et al.* recently showed that toxicity of soluble oligomeric aggregates of different proteins and peptides, including Aβ correlates with the presence of hydrophobic cavities as probed by ANS binding. The correlation suggests that hydrophobic surface may be a common feature of pathogenic protein aggregates [30], which may allow them to confer toxicity by direct interactions with membranes and/or membrane proteins.

**Figure 4:**
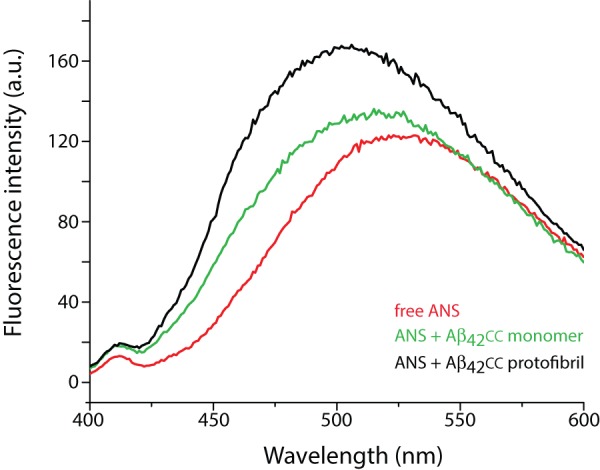
Aβ_42_ cc protofibrils expose binding sites for the ANS dye. Fluorescence emission spectra of 50 µM free ANS (red) and of ANS in the presence of Aβ_42_ cc protofibrils (black) or monomeric Aβ_42_ cc (green). Peptide concentrations are in both cases 10 µM monomer units.

We analyzed ANS binding to protofibrillar and monomeric species of Aβ_42_ cc (Fig 4). The increased fluorescence quantum yield of ANS and a blue shift of the emission spectrum from 525 to 500 in the presence of Aβ_42_ cc protofibrils suggest that hydrophobic ANS-binding sites form on the surface of Aβ_42_ cc protofibrils and that Aβ_42_ cc protofibrils are similar to other toxic protein oligomers in this regard [30]. Similar, but smaller, changes in the ANS emission can be observed with monomeric Aβ_42_ cc, but it is not clear if monomeric Aβ_42_ cc also binds ANS or if small amounts of Aβ_42_ cc aggregates are present also in these samples.

### Aβ_42_cc protofibrils share conformational epitopes with wild type Aβ oligomers

We previously reported [16] that toxic β-sheet containing oligomers and/or protofibrils of Aβcc are recognized by the mAb158 monoclonal antibody, which was selected based on its affinity for protofibrils of wild type Aβ [31]. Aβcc protofibrils are on the other hand not recognized by the A11 serum, which recognizes wild type Aβ prefibrillar oligomers as well as oligomers of other peptides [32]. However, smaller oligomers of Aβ_40_ cc with less developed β-sheet content may avoid the protofibrillar state upon further aggregation and instead form aggregates that are indeed recognized by A11 [16]. This and other observations led us to suggest that Aβcc, like wild type Aβ, aggregates along at least two pathways [16].

The question remains, however, whether the aggregation pathways followed by Aβcc actually also correspond to wild type aggregation pathways. We used the OC serum to address this issue. OC was obtained following immunization by fibrillar Aβ, but OC recognizes an epitope that is common to amyloid fibrils and certain Aβ oligomers [21], [33]. Glabe *et al.* used the OC serum to show that Aβ forms two immunologically distinct types of oligomers along two aggregation pathways: “pre-fibrillar” oligomers that are recognized by A11, but not by OC, and “fibrillar” oligomers that are recognized by OC, but not by A11 [21], [33].

**Figure 5:**
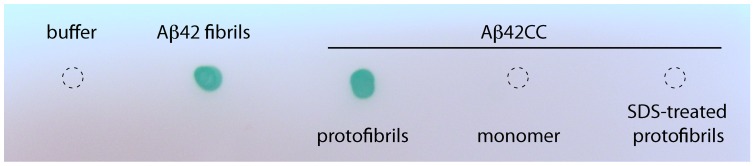
OC serum dot blot. The fibril specific OC serum recognizes Aβ_42_ cc protofibrils and wild type Aβ_42_ fibrils, but not monomeric Aβ_42_ cc or protofibrils that have been denatured by boiling in SDS.

We performed dot blot assays for OC binding to Aβ_42_ cc protofibrils and monomeric Aβ_42_ cc that had been immobilized on nitrocellulose membranes. The OC serum recognizes Aβ_42_ cc protofibrils and wild type Aβ_42_ fibrils to the same extent in this assay, whereas no binding can be detected to monomeric Aβ_42_ cc (Fig. 5). The assay is conformation specific because SDS-treated Aβ_42_ cc protofibrils are not recognized by OC (Fig. 5). These results place Aβ_42_ cc protofibrils in the same category of aggregated species as the fibrillar oligomers formed along the A11 negative/OC positive aggregation pathway of wild type Aβ.

### Aβ_42_cc protofibrils bind Apolipoprotein E in human serum

**Figure 6:**
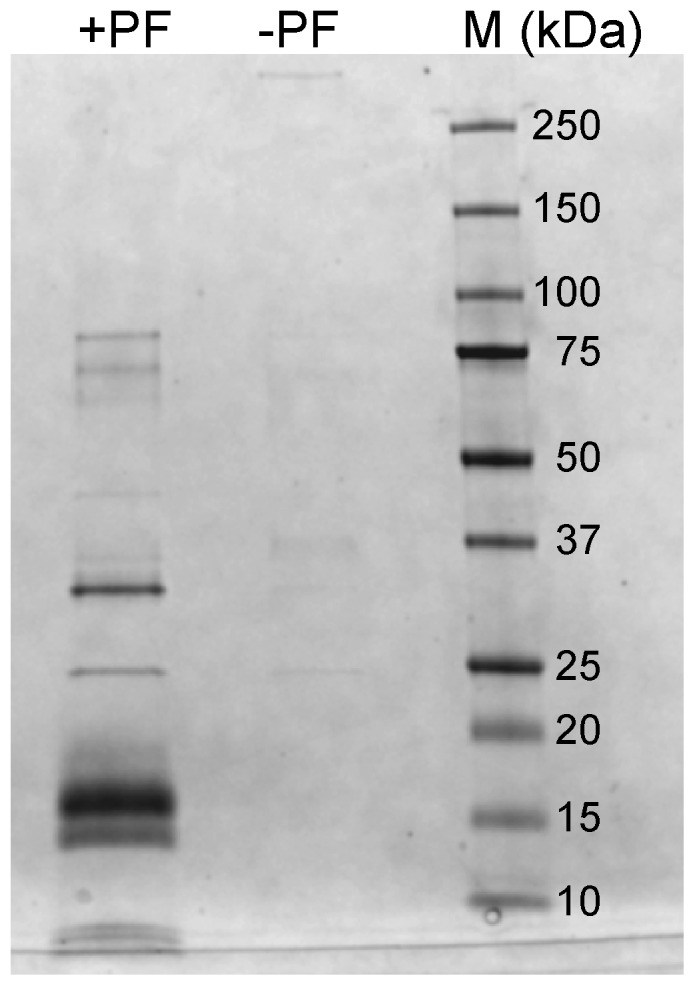
SDS-PAGE showing the separation of protein interaction partners of Aβ_42_ cc protofibrils (PF) extracted from human serum (M  =  molecular mass markers). The arrow indicates the band corresponding to apolipoprotein E. Essentially no binding is observed in control experiments with glycine-coated beads (-PF). The strong bands around 15 kDa are SDS-stable dimers and trimers of Aβ_42_ cc, as reported previously [16].

The high stability of Aβ_42_ cc protofibrils makes them potentially useful for studies of biological processes related to AD. To test this possibility we performed a pilot pull-down study to identify protein binders in biological fluids. Using Aβ_42_ cc protofibrils immobilized on magnetic beads we were able to extract protein ligands from human serum (Fig 6). Interestingly, the strongest (or most prevalent) binder was identified as apolipoprotein E (isoform ApoE4; 67% sequence coverage and Mascot score  = 348). Several other proteins were also identified and a complete analysis of the data will be presented elsewhere. ApoE is a ubiquitous cholesterol-binding protein that is linked to Aβ biology and plaque deposition and the ApoE4 isoform is a genetic risk factor for AD [1]. ApoE has also been shown to interfere with Aβ aggregation and to stabilize oligomeric forms [34]. The identification of ApoE4 as a binder to Aβcc protofibrils in serum therefore supports the relevance of these as an engineered model of wild type Aβ protofibrils and suggests that Aβ_42_ cc protofibrils may be employed in proteomics to identify interacting proteins.

### Synaptotoxicity of Aβ_42_cc protofibrils

We previously assessed the toxicity of Aβcc aggregates by measuring their effect on the rate of apoptosis in SH-SY5Y neuroblastoma cells. We found that β-sheet containing oligomers and/or protofibrils of Aβ_42_ cc induced a dose-dependent apoptosis to an extent that equaled, or perhaps exceeded, that of wild type oligomer preparations. Monomeric Aβcc or low-molecular weight oligomers of Aβ_42_ cc or monomeric or fibrillar Aβ_42_ did not, on other hand, show any effects on apoptosis in the studied concentration range. This assay confirmed toxicity, but it would be desirable to monitor the more relevant effects on synaptic activity of living neurons in a more sophisticated assay.

**Figure 7:**
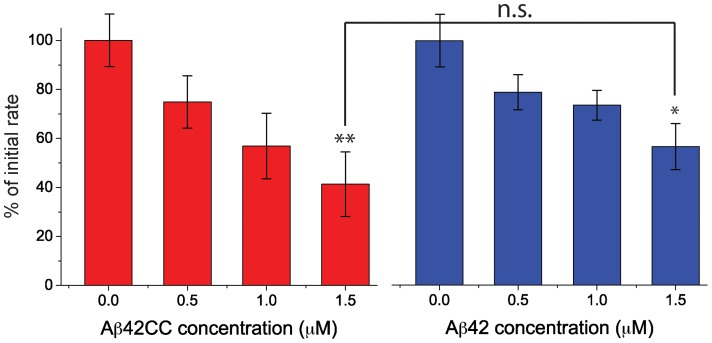
Effect of Aβ_42_ cc protofibrils (red) and wild type Aβ_42_ oligomers (blue) on spontaneous synaptic activity in mouse primary hippocampal neurons grown on a multielectrode array (MEA) chip. Changes in firing rates are normalized to the initial electrical activity in the absence of treatment and compared to buffer-treated neurons: ** – p<0.0015, * – p<0.026 (Student's t-test); the difference between Aβ_42_ oligomers and Aβ_42_ cc protofibrils is not significant.

Therefore, we analyzed the influence of Aβ_42_ cc protofibrils on synaptic activity in primary mouse hippocampal neurons cultured on the surface of microelectrode array chips, which allow the recording of spontaneous neuronal firing [9]. For comparison in this experiment we used oligomers of wild type Aβ_42_ prepared as in previous applications of this neuronal activity assay, but in the same phosphate buffer as the Aβ_42_ cc protofibrils. Treatment with 1.5 µM of either Aβ_42_ cc protofibrils or wild type Aβ_42_ oligomers both significantly inhibited spontaneous neuronal activity as compared to buffer-treated culture; the Student's t-test **p<0.0015 and *p<0.026, respectively (Fig. 7). The effect is concentration dependent and the toxicity of Aβ_42_ cc protofibrils is similar to that of the wild type Aβ_42_ oligomers. Aβ_42_ cc protofibrils therefore have an effect on synaptic activity that is comparable to what one would anticipate from biologically relevant aggregates used in previous studies of wild type Aβ. (But the outcome of the experiment does not exclude the possibility that the Aβ_42_ oligomers used for comparison are morphologically different from the Aβ_42_ cc protofibrils.)

### Summary: Aβ_42_cc protofibrils as a stable mimic of wild type protofibrils

Protofibrils were the first soluble aggregates of Aβ to be observed [24], [35], and their neurotoxicity was reported shortly thereafter [8]. Focus on protofibrils was further motivated by AD genetics since the Arctic Glu22Gly mutation in Aβ which is associated with early on-set AD, results in an increased rate of protofibril formation [36]. Protofibrils form readily *in vitro* and they are easily prepared from solubilized Aβ by size exclusion chromatography [12]. However, they convert into amyloid fibrils; 20 µM samples of Aβ_42_ form fibrils within a few hours of preparation in physiological buffer at room temperature [37]. Protofibrils may be kept at longer times under alkaline conditions [38]. However, preparations that are stable at physiological pH would have a range of applications in for instance cell biological assays and immunotherapeutic applications.

Aβcc was engineered to form hairpin conformations that are closed by an intramolecular disulfide bond between Cys21 and Cys30, which replace wild type Ala21 and Ala30. The motivation for this particular intramolecular linkage came from the observation of a corresponding hairpin of Aβ in complex with an Affibody binding protein [18], [39] and from a number of studies that indicate a propensity for such conformations in monomeric Aβ [40], [41], [42], [43]. We had, in connection to these observations, also suggested that the hairpin form of Aβ is present in oligomeric aggregates, and it was subsequently also identified in soluble aggregates [44].

The initial characterization showed that Aβ_40_ cc and Aβ_42_ cc form soluble oligomeric and protofibrillar aggregates with properties similar to those formed by wild type Aβ [16]. The aggregation occurs along two pathways that can be distinguished using the oligomer specific A11 serum and the mAb158 monoclonal antibody, respectively [16]. Aβ_40_ cc has a tendency to form aggregates recognized by the A11 serum. Aβ_42_ cc, on the other hand, spontaneously aggregates along a pathway that involves formation of anti-parallel β-sheet secondary structure, which is also present in wild type Aβ aggregates [45], to form protofibrils that are morphologically indistinguishable from wild type protofibrils when observed in electron microscopy. Aggregates formed along this “β-sheet” pathway are recognized by the mAb158 antibody, but not by the A11 serum. We found that they contain SDS-resistant oligomeric building blocks, with the same stoichiometry as in the SDS-stable aggregates of Aβ that are directly associated with AD [46], and that they are powerful inducers of apoptosis in the SH-SY5Y neuroblastoma cell line, which is not the case for monomeric or fibrillar peptide species.

However, unlike wild type, Aβcc cannot form amyloid fibrils unless the intramolecular Cys21–Cys30 disulfide bond is broken by a reduction agent such as TCEP [16]. Aβ_42_ cc, in particular, instead enriches into stable protofibrils. In this work we performed a number of complementary experiments to characterize these protofibrils in more detail. First, we examine Aβ_42_ cc protofibrils using atomic force microscopy (AFM), analytical ultracentrifugation (AUC), and nanoparticle tracking analysis (NTA) to define their morphology (rod-like) and length (60 to 220 nm on dry mica; even longer in solution). Second, we find that Aβ_42_ cc protofibrils bind the fluorescent dye ANS and therefore share surface properties that are common to cytotoxic protein aggregates including those of wild type Aβ_42_ [30]. Third, we complement previous studies of A11 serum and mAb158 antibody binding with measurements of OC serum [21] recognition to find that Aβ_42_ cc protofibrils exhibit the conformational (immunological) properties that also distinguish fibrillar oligomers of wild type Aβ_42_ from A11 binding prefibrillar oligomers [21], [33]. Fourth, the biologically relevance of the Aβ_42_ cc is further strengthened by the observed binding to apolipoprotein E in human serum. Finally, we find that Aβ_42_ cc protofibrils are not only “toxic” but also specifically affect spontaneous synaptic activity in a neuronal cell assay.

## Conclusions

We present a detailed characterization of the protofibrils that form when Aβ_42_ is stabilized in a hairpin conformation in Aβ_42_ cc. The experiments must not be interpreted as evidence for protofibrils as the most relevant form of biologically active Aβ species. Neither can they be seen as a completely unambiguous comparison with wild type protofibrils, because such a comparison is, for instance, complicated by the range of aggregates with different size and morphology that potentially can form in any sample of Aβ_42_. Nevertheless, the characteristics of Aβ_42_ cc aggregates indicate that the major determinant of Aβ toxicity is conformation, and that this conformation differs markedly from the cross-β conformation found in fibrillar Aβ. In conclusion, based on the multi-faceted coherence between Aβ_42_ cc and wild type Aβ_42_ aggregates that we observe here and reported previously [16] we suggest that the protofibrillar form of Aβ_42_ cc is a close chemical and structural mimic to the protofibrils formed by wild type Aβ.

## Supporting Information

Figure S1 **AFM image of a long Aβ_42_cc protofibril.**
(TIFF)

Figure S2 **AFM image of transiently formed aggregates in a wild type Aβ_42_ aggregation reaction mixture.** (A) and (B) show the same AFM image with different contrasting. Bundles of Aβ_42_ fibers, single fibers (blue arrow) and amorphous aggregates (green circle) can be observed in (A), and (B) reveals the presence of spherical oligomers (yellow circles) and protofibrils (red arrows). The sample was prepared by incubating ∼100 µM Aβ_42_ monomer without shaking at room temperature for one day followed by overnight incubation at 37°C with shaking.
(PDF)

## References

[1] Querfurth HW, LaFerla FM (2010) Alzheimer's disease. N Engl J Med 362: 329–344. 20107219 10.1056/NEJMra0909142

[2] Hepler RW, Grimm KM, Nahas DD, Breese R, Dodson EC, et al (2006) Solution state characterization of amyloid β-derived diffusible ligands. Biochemistry 45: 15157–15167. 17176037 10.1021/bi061850f

[3] Lambert MP, Barlow AK, Chromy BA, Edwards C, Freed R, et al (1998) Diffusible, nonfibrillar ligands derived from Aβ1-42 are potent central nervous system neurotoxins. Proc Natl Acad Sci USA 95: 6448–6453. 9600986 10.1073/pnas.95.11.6448 PMC27787

[4] Lesné S, Koh MT, Kotilinek L, Kayed R, Glabe CG, et al (2006) A specific amyloid-β protein assembly in the brain impairs memory. Nature 440: 352–357. 16541076 10.1038/nature04533

[5] Martins IC, Kuperstein I, Wilkinson H, Maes E, Vanbrabant M, et al (2008) Lipids revert inert Aβ amyloid fibrils to neurotoxic protofibrils that affect learning in mice. EMBO J 27: 224–233. 18059472 10.1038/sj.emboj.7601953 PMC2206134

[6] Ono K, Condron MM, Teplow DB (2009) Structure-neurotoxicity relationships of amyloid β-protein oligomers. Proc Natl Acad Sci USA 106: 14745–14750. 19706468 10.1073/pnas.0905127106 PMC2736424

[7] Shankar GM, Li S, Mehta TH, Garcia-Munoz A, Shepardson NE, et al (2008) Amyloid-β protein dimers isolated directly from Alzheimer's brains impair synaptic plasticity and memory. Nat Med 14: 837–842. 18568035 10.1038/nm1782 PMC2772133

[8] Hartley DM, Walsh DM, Ye CP, Diehl T, Vasquez S, et al (1999) Protofibrillar intermediates of amyloid β-protein induce acute electrophysiological changes and progressive neurotoxicity in cortical neurons. J Neurosci 19: 8876–8884. 10516307 10.1523/JNEUROSCI.19-20-08876.1999 PMC6782787

[9] Kuperstein I, Broersen K, Benilova I, Rozenski J, Jonckheere W, et al (2010) Neurotoxicity of Alzheimer's disease Aβ peptides is induced by small changes in the Aβ42 to Aβ40 ratio. EMBO J 29: 3408–3420. 20818335 10.1038/emboj.2010.211 PMC2957213

[10] Jan A, Adolfsson O, Allaman I, Buccarello A-L, Magistretti PJ, et al (2011) Aβ42 neurotoxicity is mediated by ongoing nucleated polymerization process rather than by discrete Aβ42 species. J Biol Chem 286: 8585–8596. 21156804 10.1074/jbc.M110.172411 PMC3048741

[11] Wogulis M, Wright S, Cunningham D, Chilote T, Powell K, et al (2005) Nucleation-dependent polymerization is an essential component of amyloid-mediated neuronal cell death. J Neurosci 21: 1071–1080. 10.1523/JNEUROSCI.2381-04.2005 PMC6725948 15689542

[12] Jan A, Hartley DM, Lashuel HA (2010) Preparation and characterization of toxic Aβ aggregates for structural and functional studies in Alzheimer's disease research. Nat Protoc 5: 1186–1209. 20539293 10.1038/nprot.2010.72

[13] Roychaudhuri R, Yang M, Hoshi MM, Teplow DB (2009) Amyloid β-protein assembly and Alzheimer disease. J Biol Chem 284: 4749–4753. 18845536 10.1074/jbc.R800036200 PMC3837440

[14] Chiti F, Dobson CM (2006) Protein misfolding, functional amyloid, and human disease. Ann Rev Biochem 75: 333–366. 16756495 10.1146/annurev.biochem.75.101304.123901

[15] Härd T (2011) Protein engineering to stabilize soluble amyloid β-protein aggregates for structural and functional studies. FEBS J 278: 3884–3392. 21824290 10.1111/j.1742-4658.2011.08295.x

[16] Sandberg A, Luheshi LM, Söllvander S, de Barros TP, Macao B, et al (2010) Stabilization of neurotoxic Alzheimer amyloid-β oligomers by protein engineering. Proc Natl Acad Sci USA 107: 15595–19600. 20713699 10.1073/pnas.1001740107 PMC2932621

[17] Caughey B, Lansbury PT (2003) Protofibrils, pores, fibrils, and neurodegeneration: separating the responsible protein aggregates from the innocent bystanders. Ann Rev Neurosci 26: 267–298. 12704221 10.1146/annurev.neuro.26.010302.081142

[18] Hoyer W, Grönwall C, Jonsson A, Ståhl S, Härd T (2008) Stabilization of a β-hairpin in monomeric Alzheimer's amyloid-β peptide inhibits amyloid formation. Proc Natl Acad Sci USA 105: 5099–5104. 18375754 10.1073/pnas.0711731105 PMC2278213

[19] Macao B, Hoyer W, Sandberg A, Brorsson A-C, Dobson CM, et al (2008) Recombinant amyloid β-peptide production by coexpression with an Affibody ligand. BMC Biotechnology 8: 82. 18973685 10.1186/1472-6750-8-82 PMC2606684

[20] Schuck P (2000) Size-distribution analysis of macromolecules by sedimentation velocity ultracentrifugation and Lamm equation modeling. Biophys J 78: 1606–1619. 10692345 10.1016/S0006-3495(00)76713-0 PMC1300758

[21] Kayed R, Head E, Sarsoza F, Saing T, Cotman CW, et al (2007) Fibril specific, conformation dependent antibodies recognize a generic epitope common to amyloid fibrils and fibrillar oligomers that is absent in prefibrillar oligomers. Mol Neurodegeneration 2: 18. 10.1186/1750-1326-2-18 PMC2100048 17897471

[22] Perkins DN, Pappin DJC, Creasy DM, Cottrell JS (1999) Probability-based protein identification by searching sequence databases using mass spectrometry data. Electrophoresis 20: 3551–3567. 10612281 10.1002/(SICI)1522-2683(19991201)20:18<3551::AID-ELPS3551>3.0.CO;2-2

[23] Goldsbury CS, Wirtz S, Müller SA, Sunderji S, Wicki P, et al (2000) Studies on the in vitro assembly of Aβ 1–40: implications for the search for Aβ fibril formation inhibitors. J Struct Biol 130: 217–231. 10940227 10.1006/jsbi.2000.4259

[24] Walsh DM, Lomakin A, Benedek GB, Condron MM, Teplow DB (1997) Amyloid β-protein fibrillogenesis. Detection of a protofibrillar intermediate. J Biol Chem 272: 22364–22372. 9268388 10.1074/jbc.272.35.22364

[25] Nichols MR, Moss MA, Reed DK, Lin W-L, Mukhopadhyay R, et al (2002) Growth of β-amyloid(1–40) protofibrils by monomer elongation and lateral association. Characterization of distinct products by light scattering and atomic force microscopy. Biochemistry 41: 6115–6127. 11994007 10.1021/bi015985r

[26] Cantor CR, Schimmel PR (1980) Biophysical Chemistry: W.H. Freeman and Company.

[27] Filipe V, Poole R, Kutscher M, Forier K, Braeckmans K, et al (2011) Fluorescence single particle tracking for the characterization of submicron protein aggregates in biological fluids and complex formulations. Pharm Res 28: 1112–1120. 21298328 10.1007/s11095-011-0374-0 PMC3073042

[28] Hawe A, Sutter M, Jiskoot W (2008) Extrinsic fluorescent dyes as tools for protein characterization. Pharm Res 25: 1487–1489. 18172579 10.1007/s11095-007-9516-9 PMC2440933

[29] Matthews C (1993) Pathways of protein folding. Ann Rev Biochem 62: 653–683. 8352599 10.1146/annurev.bi.62.070193.003253

[30] Bolognesi B, Kumita J, Barros T, Esbjörner E, Luheshi L, et al (2010) ANS binding reveals common features of cytotoxic amyloid species. ACS Chem Biol 5: 735–740. 20550130 10.1021/cb1001203

[31] Englund H, Sehlin D, Johansson A-S, Nilsson LNG, Gellerfors P, et al (2007) Sensitive detection of amyloid-β protofibrils in biological samples. J Neurochem 103: 334–345. 17623042 10.1111/j.1471-4159.2007.04759.x

[32] Kayed R, Head E, Thompson JL, McIntire TM, Milton SC, et al (2003) Common structure of soluble amyloid oligomers implies common mechanism of pathogenesis. Science 300: 486–489. 12702875 10.1126/science.1079469

[33] Glabe CG (2008) Structural classification of toxic amyloid oligomers. J Biol Chem 283: 29639–29643. 18723507 10.1074/jbc.R800016200 PMC2573087

[34] Cerf E, Gustot A, Goormaghtigh E, Ruysschaert J-M, Raussens V (2011) High ability of apolipoprotein E4 to stabilize amyloid-β peptide oligomers, the pathological entities responsible for Alzheimer's disease. FASEB J 25: 1585–1595. 21266538 10.1096/fj.10-175976

[35] Harper JD, Wong SS, Lieber CM, Lansbury PT (1997) Observation of metastable Aβ amyloid protofibrils by atomic force microscopy. Chem Biol 4: 119–125. 9190286 10.1016/s1074-5521(97)90255-6

[36] Nilsberth C, Westlind-Danielsson A, Eckman CB, Condron MM, Axelman K, et al (2001) The ‘Arctic’ APP mutation (E693G) causes Alzheimer's disease by enhanced Aβ protofibril formation. Nat Neurosci 4: 887–893. 11528419 10.1038/nn0901-887

[37] Luheshi LM, Hoyer W, de Barros TP, Härd IvD, Brorsson A-C, et al (2010) Sequestration of the Aβ peptide prevents toxicity and promotes degradation *in vivo* . PLoS Biology 8: e1000334. 20305716 10.1371/journal.pbio.1000334 PMC2838747

[38] Stroud JC, Liu C, Teng PK, Eisenberg D (2012) Toxic fibrillar oligomers of amyloid-β have cross-β structure. Proc Natl Acad Sci USA 109: 7717–7722. 22547798 10.1073/pnas.1203193109 PMC3356606

[39] Hoyer W, Härd T (2008) Interaction of Alzheimer's Aβ peptide with an engineered binding protein–thermodynamics and kinetics of coupled folding-binding. J Mol Biol 378: 398–411. 18358490 10.1016/j.jmb.2008.02.040

[40] Lam AR, Teplow DB, Stanely HE, Urbanc B (2008) Effects of the Arctic (E22–>G) mutation on amyloid β-protein folding: discrete molecular dynamics study. J Am Chem Soc 130: 17413–17422. 19053400 10.1021/ja804984h

[41] Lazo ND, Grant MA, Condron MC, Rigby AC, Teplow DB (2005) On the nucleation of amyloid β-protein monomer folding. Protein Sci 14: 1581–1596. 15930005 10.1110/ps.041292205 PMC2253382

[42] Mitternacht S, Staneva I, Härd T, Irbäck A (2010) Comparing the folding free-energy landscape of Aβ42 variants with different aggregation properties. Proteins 78: 2600–2608. 20589631 10.1002/prot.22775

[43] Sgourakis NG, Yan Y, McCallum SA, Wang C, Garcia AE (2007) The Alzheimer's peptides Aβ40 and 42 adopt distinct conformations in water: a combined MD/NMR study. J Mol Biol 368: 1448–1457. 17397862 10.1016/j.jmb.2007.02.093 PMC1978067

[44] Yu L, Edalji R, Harlan J, Holzman T, Lopez A, et al (2009) Structural characterization of a soluble amyloid β-peptide oligomer. Biochemistry 48: 1870–1877. 19216516 10.1021/bi802046n

[45] Cerf E, Sarroukh R, Tamamizu-Kato S, Breydo L, Derclaye S, et al (2009) Antiparallel β-sheet: a signature structure of the oligomeric amyloid β-peptide. Biochem J 421: 415–423. 19435461 10.1042/BJ20090379

[46] Jin M, Shepardson N, Yang T, Chen G, Walsh D, et al (2011) Soluble amyloid β-protein dimers isolated from Alzheimer cortex directly induce Tau hyperphosporylation and neuritic degeneration. Proc Natl Acad Sci USA 108: 5819–5824. 21421841 10.1073/pnas.1017033108 PMC3078381

