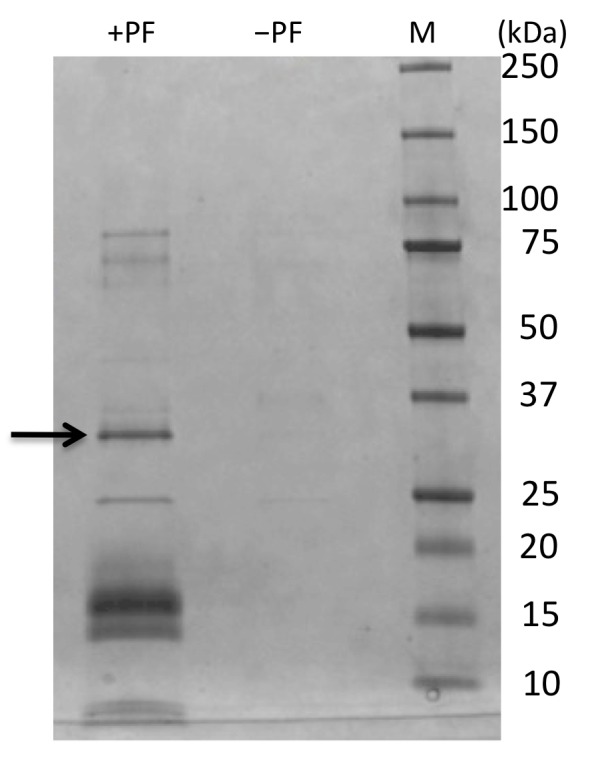# Correction: Amyloid-β Protofibrils: Size, Morphology and Synaptotoxicity of an Engineered Mimic

**DOI:** 10.1371/annotation/44be8a39-d943-419b-a430-c2b30dafadec

**Published:** 2013-10-25

**Authors:** Anatoly Dubnovitsky, Anders Sandberg, M. Mahafuzur Rahman, Iryna Benilova, Christofer Lendel, Torleif Härd

There was an error in Figure 6. The correct version of the Figure is available here: 

**Figure:**