# Supplementary figures and images for: Amyloid-β Protofibrils: Size, Morphology and Synaptotoxicity of an Engineered Mimic
Source: PLoS One. 2013 Jul 2;8(7):e66101. doi: 10.1371/journal.pone.0066101 (PMC3699592; doi:10.1371/journal.pone.0066101)

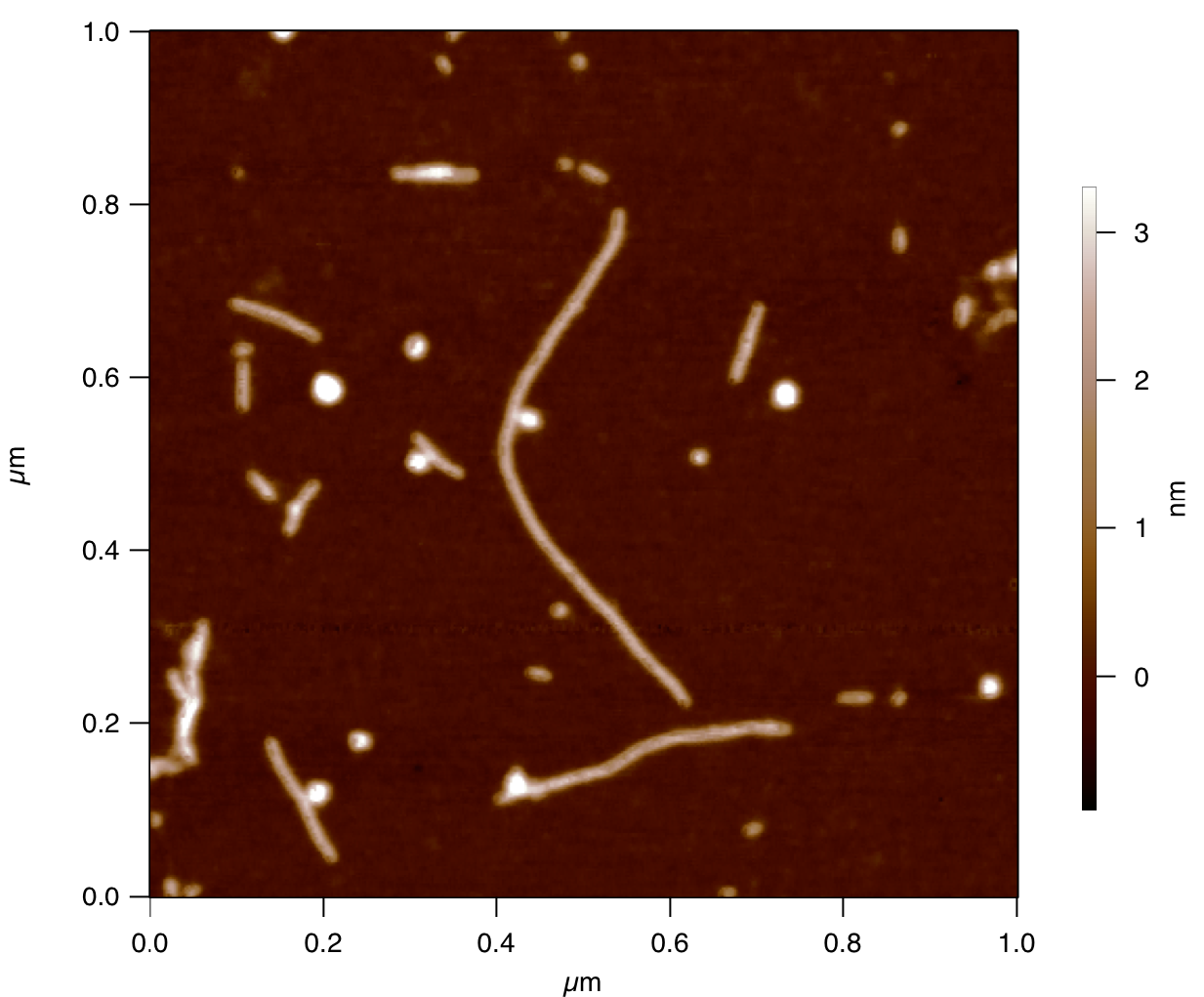

Supplement: Figure S1 — AFM image of a long Aβ42cc protofibril. (TIFF) [file pone.0066101.s001.tiff]

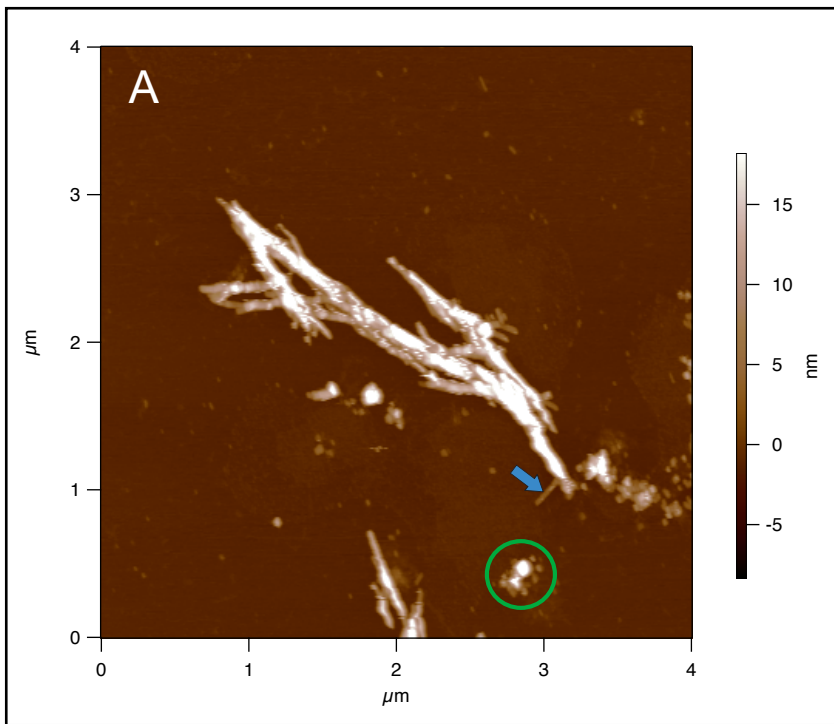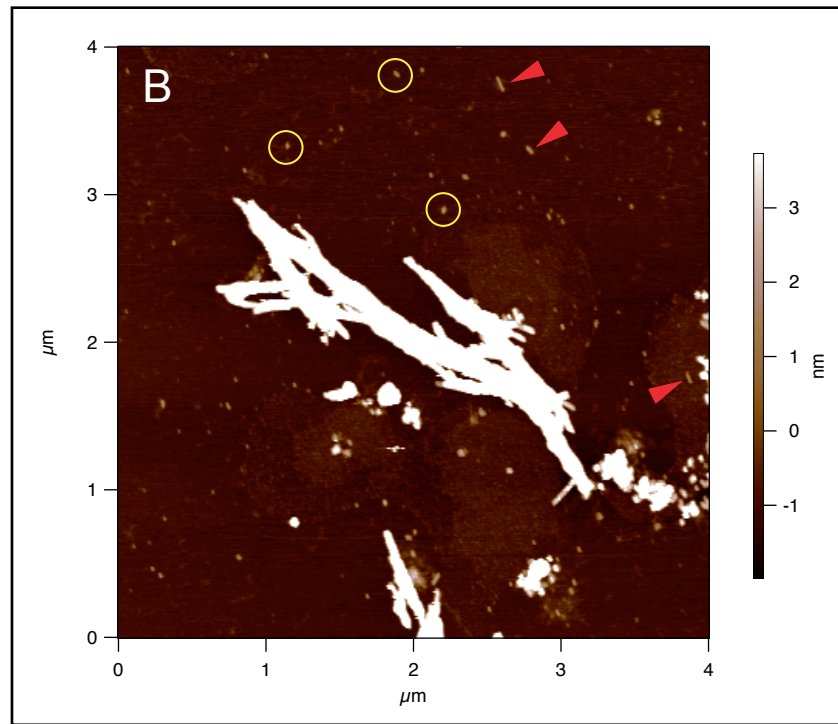

Supplement: Figure S2 — AFM image of transiently formed aggregates in a wild type Aβ42 aggregation reaction mixture. (A) and (B) show the same AFM image with different contrasting. Bundles of Aβ42 fibers, single fibers (blue arrow) and amorphous aggregates (green circle) can be observed in (A), and (B) reveals the presence of spherical oligomers (yellow circles) and protofibrils (red arrows). The sample was prepared by incubating ∼100 µM Aβ42 monomer without shaking at room temperature for one day followed by overnight incubation at 37°C with shaking. (PDF) [file pone.0066101.s002.pdf]
